# Superior antiviral activity of IFNβ in genital HSV-1 infection

**DOI:** 10.3389/fcimb.2022.949036

**Published:** 2022-10-17

**Authors:** Yasmin Schmitz, Mara Schwerdtfeger, Jaana Westmeier, Elisabeth Littwitz-Salomon, Mira Alt, Leonie Brochhagen, Adalbert Krawczyk, Kathrin Sutter

**Affiliations:** ^1^ Institute for Virology, University Medicine Essen, University of Duisburg-Essen, Essen, Germany; ^2^ Department of Infectious Diseases, West German Centre of Infectious Diseases, University Medicine Essen, Essen, Germany

**Keywords:** type I IFNs, IFNβ, herpes simplex virus-1, HSV infection, immunotherapy

## Abstract

Type I interferons (IFNs) present the first line of defense against viral infections, providing antiviral, immunomodulatory and antiproliferative effects. The type I IFN family contains 12 IFNα subtypes and IFNβ, and although they share the same receptor, they are classified as non-redundant, capable to induce a variety of different IFN-stimulated genes. However, the biological impact of individual subtypes remains controversial. Recent data propose a subtype-specificity of type I IFNs revealing unique effector functions for different viruses and thus expanding the implications for IFNα-based antiviral immunotherapies. Despite extensive research, drug-resistant infections with herpes simplex virus type 1 (HSV-1), which is the common agent of recurrent orogenital lesions, are still lacking a protective or curing therapeutic. However, due to the risk of generalized infections in immunocompromised hosts as well as the increasing incidence of resistance to conventional antiherpetic agents, HSV infections raise major health concerns. Based on their pleiotropic effector functions, the application of type I IFNs represents a promising approach to inhibit HSV-1 replication, to improve host immunity and to further elucidate their qualitative differences. Here, selective IFNα subtypes and IFNβ were evaluated for their therapeutic potential in genital HSV-1 infections. Respective *in vivo* studies in mice revealed subtype-specific differences in the reduction of local viral loads. IFNβ had the strongest antiviral efficacy against genital HSV-1 infection in mice, whereas IFNα1, IFNα4, and IFNα11 had no impact on viral loads. Based on flow cytometric analyses of underlying immune responses at local and peripheral sites, these differences could be further assigned to specific modulations of the antiviral immunity early during HSV-1 infection. IFNβ led to enhanced systemic cytokine secretion and elevated cytotoxic responses, which negatively correlated with viral loads in the vaginal tract. These data provide further insights into the diversity of type I IFN effector functions and their impact on the immunological control of HSV-1 infections.

## Introduction

As key mediators of the innate immunity, type I IFNs hold crucial functions for the defense against viral infections, by modulating a variety of cellular responses like proliferation, apoptosis or immune regulation and the establishment of an antiviral state. In humans, the type I IFN family comprises 17 functional genes for IFNβ, IFNϵ, IFNκ, IFNω and 13 IFNα subtypes (Freaney et al., 2014). Thereby, the sequence identity among type I IFNs constitutes not more than 30 %, resulting in specific activities and tissue distributions for individual subtypes (Chen et al., 2004; Thomas et al., 2011). Despite the high genetic and corresponding structural conservation of IFNα subtypes (Wittling et al., 2020), each subtype though is encoded by a single gene, assuming them to be non-redundant. Consistent with this postulation, differing antiviral and immunomodulatory activities for IFNα subtypes have been observed (Lavender et al., 2016; Dickow et al., 2019; Guo et al., 2020; Chen et al., 2021; Schuhenn et al., 2022). However, the complex system of type I IFN responses and their regulation is still not completely resolved.

The clinical administration of IFNα2 presented a potent strategy in the treatment of chronic hepatitis B virus (HBV) and hepatitis C virus (HCV) for more than three decades (Scott and Perry, 2002; Rong and Perelson, 2010; Woo et al., 2017; Ye et al., 2021). However, the biological role of individual type I IFN subtypes is mostly unresolved. In this regard, recent studies could reveal subtype-specific differences in the antiviral efficacy among type I IFNs, resulting in varying IFN-stimulated gene (ISG) expression patterns and corresponding effector functions for certain viruses.

According to this subtype-specificity, the therapeutic application of exogenous type I IFNs has regained importance as potential treatment strategy for further viruses. In this concern, HSV-1 still constitutes a major challenge of latent viral infections. Among the family of *Herpesviridae*, HSV-1 is the most frequently represented herpesvirus in humans with a worldwide seroprevalence of approx. 67 % in adults, characterized by recurrent, self-limiting lesions at oral and genital sites (Whitley and Roizman, 2001; Looker and Garnett, 2005; Fatahzadeh and Schwartz, 2007; James et al., 2020). HSV-1 presents a unique lifestyle, which is characterized by alternating phases of lytic replication and lifelong latency in sensory neurons of predominantly trigeminal ganglia (Bearer et al., 2000; Theil et al., 2003). Both primary infection and periodic reactivation from latency usually lead to formation of painful, mucocutaneous vesicles or ulcers, that crust over and heal without scarring, commonly known as *herpes labialis* or *herpes genitalis* (Spruance et al., 1977; Corey et al., 1983). Nevertheless, HSV infections can expand to further sites including liver, lung or the central nervous system, thus developing a fulminant course with in part life-threatening complications, especially in newborns and immunocompromised patients (Kimberlin, 2004; Herget et al., 2005). Until today the treatment of HSV infections still presents a major public health issue. In fact, several antiherpetic agents are commercially available. However, their efficacy is restricted to local skin lesions without reaching viral clearance and it is further limited by an increasing incidence of resistant virus mutants (Perry and Faulds, 1996; Danve-Szatanek et al., 2004; Lebrun-Vignes et al., 2007; Hammer et al., 2018; Álvarez et al., 2020).

In HSV infections, type I IFNs are known to have a crucial impact on the interplay of host defense and immune evasion, thus presenting promising candidates for antiviral treatment strategies. Following virus recognition by different pathogen-recognition receptors (Kurt-Jones et al., 2017), the production and release of endogenous type I IFNs results in a broad stimulation of several host immune cells, thus promoting the establishment of an antiherpetic environment (Hervas-Stubbs et al., 2011; Mesev et al., 2019). During acute infection, especially innate natural killer (NK) cell effector functions are stimulated by type I IFNs. Corresponding to the overall cytotoxic and cytolytic activity of NK cells in viral infections (Caligiuri, 2008), in particular their secretion of IFNγ seems to have a major impact on HSV control (Gill et al., 2011). Later during infection, type I IFN activities influence T cells with the induction of cytokine secreting CD4^+^ T cells and the stimulation of Granzyme B (GzmB) and IFNγ expression by cytotoxic CD8^+^ T cells (Daheshia et al., 1999; Liu et al., 2000; Carr and Noisakran, 2002; Knickelbein et al., 2008; Vahed et al., 2019). Besides their immunomodulatory effects, type I IFNs directly impede the replication of HSV (Austin et al., 2005; Carr et al., 2005). However, the induced immune responses are not able to prevent virus spreading and subsequent establishment of latency. Hence, HSV is predicted to hijack the host’s immune system by specific escape mechanisms, most likely due to downregulation of type I IFN signaling.

Despite these evasion mechanisms, type I IFNs still present promising candidates for HSV treatment, assuming the application of additional, extrinsic type I IFN subtypes to reinvigorate the activity of endogenous IFNs. Several *in vivo* and *in vitro* studies reported HSV-specific preventive and therapeutic effects of human and murine type I IFN subtypes, leading to a reduction in viral titers and recurrence events as well as an accelerated lesion remission, whereas local and peripheral immune responses were not investigated in detail (Jones et al., 1976; Ho, 1990; Cardamakis et al., 1998; Carr and Noisakran, 2002; Härle et al., 2002; Härle et al., 2002; Austin et al., 2006). In this regard, previous *in vivo* studies with further recombinant IFNα subtypes as well as plasmids, expressing different type I IFN genes, examined a subtype-specificity in the antiherpetic potential, thus proposing other type I IFN subtypes than human IFNα2 to be more efficient for HSV treatment (Härle et al., 2001; Austin et al., 2006; Giraldo et al., 2020).

Hence, within the present work we addressed the question whether the application of exogenous IFNα subtypes or IFNβ can overcome the HSV-specific immune restriction and show beneficial effects on the viral burden. Therefore, the therapeutic potential of selective murine type I IFNs was evaluated in an *in vivo* mouse model of genital HSV-1 infection. Respectively, the reduction of local viral loads and potential differences in the antiviral efficacy of type I IFN subtypes was elucidated. IFNβ demonstrated a superior anti-HSV-activity *in vivo* compared to the other tested IFNα subtypes. Furthermore, treatment with IFNβ improved cytotoxic T and NK cell responses at local and peripheral sites early during HSV-1 infection, which negatively correlated with viral loads at early time points in the vaginal tract. In conclusion, we identified distinct immune cell effector functions during vaginal HSV-1 infection, which strongly correlated with the immunotherapeutic potential of murine IFNβ.

## Material and methods

### Cells, mice and virus

The African green monkey kidney cell line Vero and the murine fibroblast cell line NIH-3T3 were grown in Dulbecco’s Modified Eagle’s medium (DMEM, PAN-Biotech) supplemented with 10 % heat-inactivated fetal calf serum (FCS, Sigma-Aldrich), 100 U/mL penicillin and 100 µg/mL streptomycin (Thermo Fisher Scientific). For infection experiments cells were maintained in DMEM with 2 % FCS.

Female C57BL/6 mice were purchased from Charles River Laboratories and Envigo. Mice were at least 8 weeks of age and daily monitored for their general state of health, spontaneous behavior and body weight. Mice were kept according to the guidelines and regulations of the institutional animal care and use committee of the University of Duisburg-Essen, Germany. All procedures were performed according to the German regulations of the Society for Laboratory Animal Science (GV-SOLAS) and the European Health Law of the Federation of Laboratory Animal Science Associations (FELASA). The corresponding protocol was approved by the North Rhine-Westphalia State Agency for Nature, Environment and Consumer Protection (LANUV). All efforts were made to minimize suffering.

Wild-type virus strain HSV-1 F was propagated and titrated on Vero cell monolayers. Infectious titers were determined by Vero cell-based HSV plaque assay as described below and calculated as 50 % tissue culture infectious doses TCID_50_/mL.

### Vaginal HSV-1 infections

To increase the susceptibility for genital HSV challenge, mice were pre-treated with Sayana^®^ medroxyprogesterone acetate (Pfizer) prior to intravaginal infection, thus inducing and prolonging their diestrus state. At least 7 days before infection, mice were intraperitoneally (i.p.) injected with 2.5 mg Sayana^®^ diluted in phosphate-buffered saline (PBS, Thermo Fisher Scientific). On the day of infection, mice were anesthetized by i.p. injection with 50 mg/kg ketamine and 8 mg/kg xylazine. Subsequently, the vaginal mucosa was cleaned with a sterile cotton swab and infected with 1×10^7^ TCID_50_/20 µL of HSV-1 F diluted in PBS or 20 µL of PBS for naive control mice. To allow the inoculum to infect, the vaginal opening was temporarily closed with EPIGLU^®^ skin glue (Meyer-Haake Medical Innovations).

### *In vitro* HSV-1 infection and type I IFN stimulation

NIH-3T3 cells were seeded into 24-well plates in 10 % DMEM to reach a confluency of 80-90 % the next day. Six hours later, cells were stimulated with 1000 U/mL murine IFNα subtypes or IFNβ. The next day, cultivation medium of cells was replaced by 2 % DMEM and cells were infected with 5×10^5^ TCID_50_/mL of HSV-1 F. Finally, after incubation for additional 2 days at 37 °C, virus-containing supernatants were collected for further determination of infectious titers as described below.

### Determination of infectious titers by HSV plaque assay

NIH-3T3 cells and Vero cells were grown in a 96-well format to reach a confluency of 80-90 % the next day. Next day, murine vaginal lavage liquids were serially diluted (1:10^3^-1:10^10^) on Vero cells in six replicates. Correspondingly, titration of cell supernatants from *in vitro* infection was performed on NIH-3T3 cells (1:10-1:10^10^). Evaluation of plaque formation was performed after incubation for 4 days (NIH-3T3 cells) or 5 days (Vero cells) at 37 °C by manual counting of plaque-containing wells under the microscope. Corresponding infectious titers were calculated as TCID_50_ values according to the Spearman and Kärber algorithm (Hierholzer and Killington, 1996).

### Generation of murine type I IFNs and determination of IFN concentrations

Expression of IFNα1, IFNα4, and IFNβ was performed as previously described (Gerlach et al., 2009). To produce murine IFNα11, the cell line HEK293mIFNalpha11 was cultivated as described (Bollati-Fogolin and Muller, 2005). All concentrated supernatants were tested for type I IFN activity using the murine 3T3 ISRE-Luc reporter cell line, transfected with a plasmid containing the Firefly Luciferase gene, stably integrated under control of the IFN-stimulation-response element (ISRE). After 4.5 h of type I IFN stimulation, cells were lysed and chemiluminescence was detected using the Beetle-Juice Luciferase assay Firefly (PJK). The IFN activity was calculated to the respective activity in units against commercially available recombinant mouse IFNβ and universal IFNα (PBL assay science).

### *In vivo* type I IFN treatment and vaginal lavages

Intraperitoneal injections of murine IFNα1, IFNα4, IFNα11, or IFNβ (8000 U) were daily administered to respective mice from day 2 *post infectionem* (*p.i.*) until day 7 *p.i.* for 8-days-studies or until day 3 *p.i.* for 4-days-studies.

For verification of local viral loads, vaginal lavages were performed every second day starting on day 2 *p.i.* until the end of the study. Therefore, mice were narcotized *via* inhalation anesthesia with isoflurane, vaginal mucus was removed with sterile PBS-soaked cotton swabs and 40 µL PBS were carefully resuspended within the vaginal lumen. Vaginal lavages were frozen at -80°C until determination of infectious titers.

On day 4 *p.i.* or day 8 *p.i.*, mice were sacrificed and analyzed for local and distal immune responses.

### Isolation of vaginal tract leukocytes and spleen lymphocytes

Isolation of vaginal tract leukocytes was performed as previously described with minor modifications (Jiang and Kelly, 2012). Vaginal tracts of euthanized mice were dissected from oviducts to vaginal opening and cut into fine pieces. The minced tissue was transferred into 10 mL RPMI 1640 medium (Capricorn Scientific) supplemented with 10 % FCS, 100 U/mL penicillin, 100 µg/mL streptomycin, 1 % 1 M HEPES (Thermo Fisher Scientific) and 5 µM EDTA (AppliChem), and incubated for 15 min at room temperature while constantly shaking. Afterwards, the tissue was collected *via* 70 µm cell strainers (Miltenyi Biotec), the flowthroughs were discarded and the EDTA washing step was repeated. Afterwards EDTA was washed off with EDTA-free RPMI medium and the tissue was transferred into 3 mL RPMI medium supplemented with 10 % FCS, 100 U/mL penicillin, 100 µg/mL streptomycin, 1 % 1 M HEPES and 1 µg/mL deoxyribonuclease I (DNase, AppliChem). For subsequent digestion, 2 mg/mL collagenase IV (Sigma Aldrich) was added and the tissue suspensions incubated for 1 h at 37°C while constantly shaking. This step was repeated twice and leukocyte-containing flowthroughs were collected *via* 70 µm cell strainers, stored on ice and the remaining tissue pieces were transferred into fresh RPMI-DNase plus collagenase IV media for further digestion. Finally, collected flowthroughs were pooled and centrifuged for 7 min at 300 × g and stored at 4 °C until flow cytometry staining.

For the isolation of spleen lymphocytes, dissected spleens were mechanically dispersed into 10 mL PBS. Cell suspensions were centrifuged for 7 min at 300 × g and resulting cell pellets dissolved in an appropriate volume of PBS. Until further flow cytometry staining, prepared spleen cell suspensions were stored at 4°C.

### Cell surface and intracellular staining for flow cytometry

Cell surface and intracellular staining of vaginal tract leukocytes and spleen lymphocytes was performed as previously described (Zelinskyy et al., 2006; Zelinskyy et al., 2009). For the assessment of splenic cytokine secretion, spleen lymphocytes were re-stimulated with 25 ng/mL phorbol myristate acetate (PMA, Invivogen) and 0.5 µg/mL ionomycin (Invivogen) for 1 h at 37°C. Produced cytokines were afterwards retained by adding 2 µg/mL brefeldin A (BFA, BioLegend) for further 2 h at 37°C. Vaginal tract leukocytes were stained for CD3 (clone: 145-2C11), CD4 (GK1.5), CD8 (53-6.7), CD11b (M1/70), CD11c (N418), CD19 (6D5), CD62L (MEL-14), CD69 (H1.2F3), CD80 (16-10A1), Ki67 (SolA15), MHC-II (M5/114.15.2), NK1.1 (PK136), Perforin (eBioO-MAK-D) and TRAIL (TNF-related apoptosis-inducing ligand, N2B2), while staining of spleen lymphocytes was performed with antibodies targeting CD3 (145-2C11), CD4 (GK1.5), CD8 (53-6.7), CD62L (MEL-14), CD69 (H1.2F3), GzmB (clone GB11), IFNγ (AN.18.17.24), IL-2 (JES-6-5H4), Ki67 (SolA15), NK1.1, Perforin (eBioO-MAK-D), TNFα (MP6-XT22) and TRAIL (N2B2). Exclusion of dead cells was provided by fixable viability dye (FVD, Thermo Fischer Scientific). Fluorescence minus one controls were prepared for all conditions. FACS measurements were performed with BD™ LSR II; BD FACSymphony™ A5, or BD FACSCanto™ II flow cytometer (Becton Dickinson) and resulting data were analyzed using FlowJo™ software (Becton Dickinson). The gating strategy and representative dot plots are shown in **Supplementary Figure 2**.

### Multiplex cytokine and chemokine bead arrays

Cytokine quantification of vaginal lavages from HSV-1-infected mice (4 days post infection (dpi)) was done using the customized LEGENDplex multiplex assay (BioLegend) for the detection of the following cytokines and chemokines: CCL5 (RANTES), IFNα, IFNγ, IL-10, IL-12p40, IL-12p70, IL-15, IL-18, IL-2, IL-6, TNFα, GM-CSF, CCL3 (MIP-1α), and IL-27. The assay was performed according to the provider’s instructions and analyzed using the dedicated software provided by BioLegend. Data acquisition was performed on a BD LSRII flow cytometer.

### Statistical analysis

Experimental data were indicated as median or mean ± SEM. Statistically significant differences compared to the first day of treatment for individual groups or between infected and IFN-treated groups were analyzed using the nonparametric Kruskal-Wallis test or the 2way ANOVA and Tukey’s multiple comparison test. Statistical analyses were conducted using the GraphPad Prism software (GraphPad).

## Results

### IFNβ treatment led to reduced HSV-1 titers in vaginal tract

**Figure 1:**
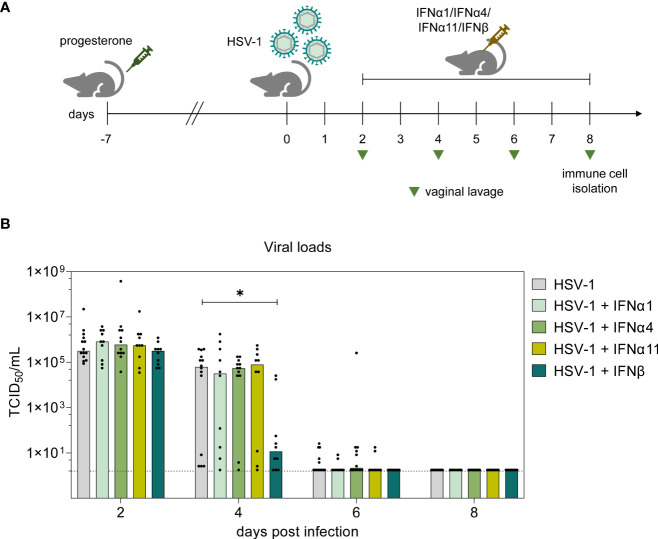
Antiviral activity of type I IFNs during vaginal HSV-1 infection on day 8 *p.i.* **(A)** Experimental setup for the *in vivo* assessment of type I IFN activities in genital HSV-1 infections. Female C57BL/6 mice were pretreated with 2.5 mg medroxyprogesterone acetate one week before infection. For intravaginal infection, mice were inoculated with 1×10^7^ TCID_50_ of HSV-1 F or left uninfected (naive control). Infected mice were assigned to different treatment groups (IFNα1, IFNα4, IFNα11, IFNβ) receiving daily injections of respective murine type I IFN subtypes (8000 U) for six consecutive days starting on day 2 *p.i.* or left untreated (HSV-1). From day 2 *p.i.* until day 8 *p.i.*, liquids from vaginal tract lavages were collected every second day for determination of viral loads, shown by light green triangles. After 8 days, spleens and vaginal tracts were isolated for further analysis of immune responses. **(B)** Viral titers of vaginal lavages were determined by endpoint dilution assay. Bars indicate the median virus titer of each group (HSV-1 *n* = 14 mice, HSV-1 + IFNα1 *n* = 10 mice, HSV-1 + IFNα4 *n* = 12 mice, HSV-1 + IFNα11 *n* = 10 mice, HSV-1 + IFNβ *n* = 10 mice). Dashed line marks the detection limit. Statistical analyses were conducted with the nonparametric Kruskal-Wallis test. Statistical significance between individual study groups is represented by *P < 0.05.

As major part of the innate immunity, type I IFNs hold a crucial impact on the control of viral infections either by direct effects on infected and neighboring cells or by modulation of associated immune cell activities (Hervas-Stubbs et al., 2011; Teijaro, 2016). Thereby, type I IFNs and especially closely related IFNα subtypes seem to hold a subtype-specificity for certain viruses (Gibbert et al., 2013; Lavender et al., 2016; Dickow et al., 2019). Type I IFN subtypes also contribute to the host defense against HSV-1, hence preventing a systemic spread of the infection. Here, we aim to decipher the underlying antiviral and immunomodulatory properties of different murine type I IFNs during HSV-1 infection as well as to identify qualitative differences between type I IFN subtypes. Murine IFNα1, IFNα4, IFNα11 and IFNβ were selected due to their previously reported antiviral potential against Friend retrovirus and HSV-2 *in vivo* (Austin et al., 2006; Gerlach et al., 2009; Gibbert et al., 2012). According to the experimental design illustrated in **Figure 1A**, one week prior to intravaginal infection female C57BL/6 mice were treated with medroxyprogesterone acetate in order to induce and prolong the diestrus state in mice, thus increasing their susceptibility for genital HSV challenge (Kaushic et al., 2003; Gillgrass et al., 2005). Following HSV-1 infection, mice were assigned to different IFN treatment groups, comprising daily injections of respective type I IFN subtypes from day 2 *p.i.* until day 7 *p.i.* The impact of IFN treatment was assessed by determining viral titers from vaginal lavages on days 2, 4, 6, and 8 *p.i.* On day 2 prior to IFN treatment, all mice showed similar initial viral loads (**Figure 1B**). Overall, a decrease of viral titers was observed on day 4 *p.i.* until day 8 *p.i.* At the end of experiments all mice had viral loads below the detection limit. Interestingly, already on day 4 *p.i*. the group of mice treated with IFNβ had more than 5400-fold lower viral titers compared to untreated controls [median TCID_50_/mL: 6.27 x 10^4^ (HSV-1) and 11.58 (HSV-1 + IFNβ)]. All other applied IFN subtypes did not show any significant effect on HSV-1 replication (**Figure 1B**), although similar antiherpetic efficacies of those IFNs were observed *in vitro* (**Supplementary Figure 1**). Also, treatment with very high concentrations of IFNα4 (40,000 units per application) did not significantly reduce viral loads on day 4 *p.i.* indicating that the lack of antiviral activity cannot be overcome by increasing concentrations (data not shown). These data nicely show qualitative rather than quantitative differences between the type I IFN subtypes during vaginal HSV-1 infection.

### IFN treatment did not improve immune responses in spleen and vaginal tract at 8 days post HSV-1 infection

**Figure 2:**
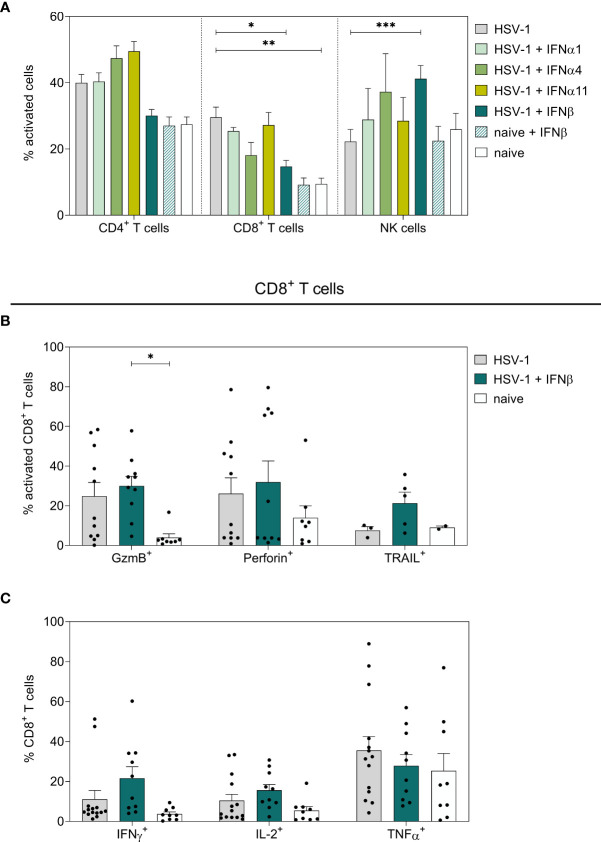
Peripheral immune responses in the spleen on day 8 post HSV-1 infection. Female C57BL/6 mice were infected with 1×10^7^ TCID_50_ of HSV-1 F for eight days or left uninfected (naive control). Mice were treated with 8000 U of murine IFNα1, IFNα4, IFNα11, and IFNβ for six consecutive days starting on day 2 *p.i.* or left untreated (HSV-1). On day 8 *p.i.*, splenic lymphocytes were isolated from dissected spleens. For flow cytometric analysis, isolated cells were subsequently stained for respective cell surface and intracellular markers. **(A)** Frequencies of activated CD4^+^ T cells, CD8^+^ T cells, and NK cells as well as **(B)** respective percentage of cytotoxic CD8^+^ T cells and **(C)** cytokine-secreting CD8^+^ T cells are shown. Data points represent individual mice; bars indicate the mean values ± SEM for each group (HSV-1 *n* = 3-14 mice, HSV-1 + IFNα1 *n* = 5 mice, HSV-1 + IFNα4 *n* = 3-5 mice, HSV-1 + IFNα11 *n* = 6-8 mice, HSV-1 + IFNβ *n* = 5-10 mice, naive + IFNβ *n* = 4 mice, naive *n* = 2-9 mice). Statistical analyses were conducted *via* 2way ANOVA and Tukey’s multiple comparison test. Statistically significant differences among all study groups are depicted by *P < 0.05, **P < 0.01, ***P < 0.001.

To scrutinize potential correlations of the observed reduction in viral loads and IFN-mediated differences in immune responses, splenocytes of IFN-treated and untreated HSV-1-infected mice were analyzed on day 8 *p.i*. As shown in **Figure 2A**, HSV-1 significantly increased the percentages of activated CD62L^-^ splenic CD8^+^ T cells, whereas daily applications of IFNβ significantly reduced these frequencies. All other tested IFNs had no significant effect on the activation of T cells. Frequencies of activated CD69^+^ NK cells were not increased in HSV-1-infected animals, but the treatment solely with IFNβ significantly elevated frequencies of CD69^+^ NK cells. In contrast, frequencies of activated CD62L^-^ CD8^+^ T cells expressing the death receptor ligand TRAIL (**Figure 2B**) and IFNγ (**Figure 2C**) slightly increased upon IFNβ immunotherapy, but this effect was not significant. Furthermore, proportions of TRAIL-expressing CD4^+^ T cells and NK cells were elevatedupon IFNβ treatment in comparison to untreated, HSV-1-infected mice (**Supplementary Figure 3**). Treatment of uninfected mice with IFNβ did not modulate immune responses as detected for IFNβ-treated HSV-1-infected mice (**Figure 2A**; **Supplementary Figure 3**). Again, application of IFNα1, α4, or α11 did not markedly affect immune responses in the spleen (**Supplementary Figure 3**).

**Figure 3:**
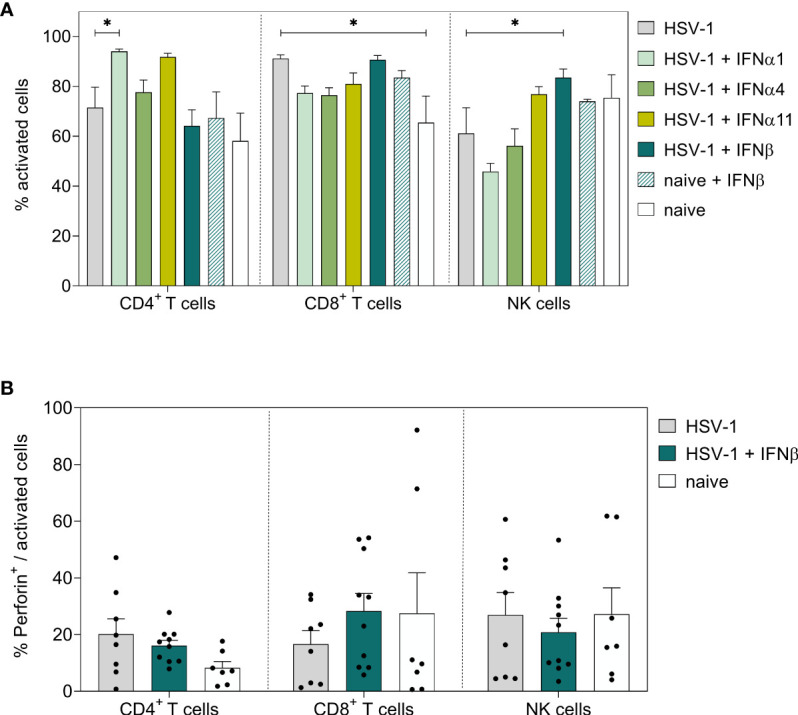
Local immune responses in the vaginal tract on day 8 post HSV-1 infection. Female C57BL/6 mice were infected with 1×10^7^ TCID_50_ of HSV-1 F for eight days or left uninfected (naive control). Mice were treated with 8000 U of murine IFNα1, IFNα4, IFNα11, and IFNβ for six consecutive days starting on day 2 *p.i.* or left untreated (HSV-1). On day 8 *p.i.*, vaginal lymphocytes were isolated from dissected vaginal tracts. For flow cytometric analysis, isolated cells were subsequently stained for respective cell surface and intracellular markers. **(A)** Frequencies of activated CD4^+^ T cells, CD8^+^ T cells, and NK cells and **(B)** percentage of respective Perforin-expressing, activated cell populations are shown. Data points represent individual mice; bars indicate the mean values ± SEM for each group (HSV-1 *n* = 8 mice, HSV-1 + IFNα1 *n* = 10 mice, HSV-1 + IFNα4 *n* = 10 mice, HSV-1 + IFNα11 *n* = 5 mice HSV-1 + IFNβ *n* = 10 mice, naive + IFNβ *n* = 4 mice, naive *n* = 7 mice). Statistical analyses were conducted *via* 2way ANOVA and Tukey’s multiple comparison test. Statistically significant differences compared to the HSV-1 group are depicted by *P < 0.05.

To further dissect the immunomodulatory effects of type I IFNs on local immune responses at the vaginal tract, vaginal lymphocytes were isolated on day 8 *p.i*. and respective cell subsets were examined for their activation and effector functions. No significant effects on activation or effector functions of NK cells (**Figure 3**, **Supplementary Figure 3G**), T cells (**Figure 3**, **Supplementary Figure 3G**), B cells (data not shown), or dendritic cells (DCs, data not shown) were observed in the vaginal tract of type I IFN-treated mice compared to untreated, infected mice. Only frequencies of Perforin-expressing CD8^+^ T cells were significantly enhanced after IFNα11-therapy; however, this treatment had no significant effect on virus control (**Figure 1B**). Taken together, compared to solely HSV-1-infected mice, type I IFN treatment had no remarkable effect on T and NK cells in the spleen and vaginal tract at day 8 post HSV-1 infection.

### Frequencies in TRAIL-expressing immune cells negatively correlated with viral loads at 4 dpi

**Figure 4:**
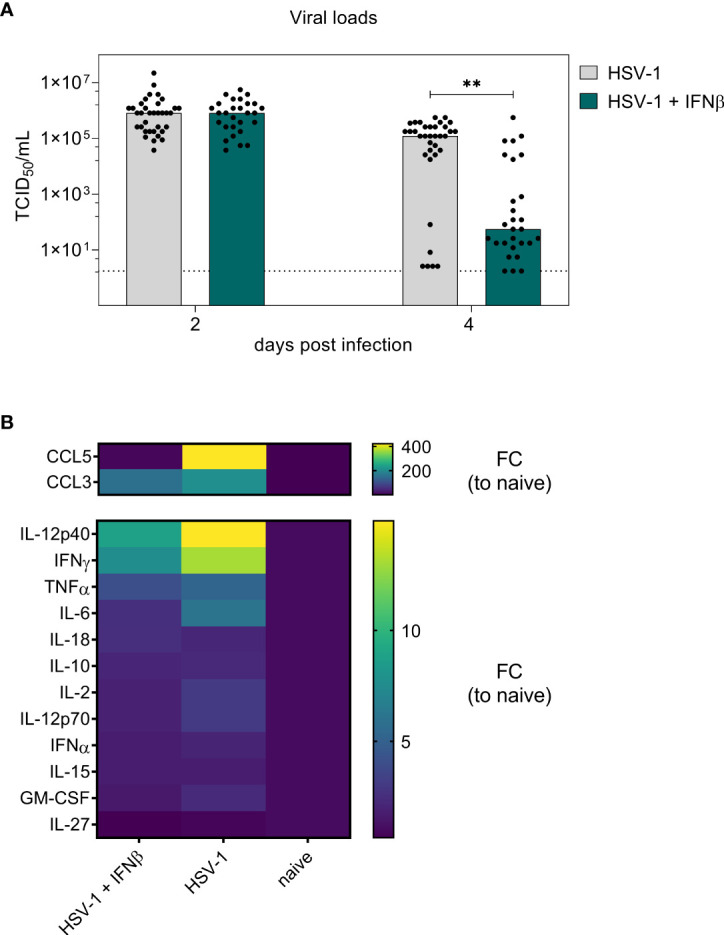
Early antiviral activity of IFNβ on day 4 post vaginal HSV-1 infection. Female C57BL/6 mice were infected with 1×10^7^ TCID_50_ of HSV-1 F for four days or left uninfected (naive control). Mice were treated with 8000 U of murine IFNβ for two consecutive days starting on day 2 *p.i.* or left untreated (HSV-1). On day 2 *p.i*. and day 4 *p.i*., liquids from vaginal tract lavages were collected for determination of viral loads. After 4 days, spleens and vaginal tracts were isolated for further analysis of immune responses. **(A)** Viral titers of vaginal lavages were determined by endpoint dilution assay. Bars indicate the median virus titer of each group (HSV-1 *n* = 35 mice, HSV-1 + IFNβ *n* = 28 mice). Dashed line represents the detection limit. Statistically significant differences of mean HSV-1 + IFNβ titers compared to the initial viral load in infected mice were analyzed with the nonparametric Kruskal-Wallis test and are depicted by **P < 0.01. **(B)** Fold change (FC) of CCL5 (RANTES), IFNα, IFNγ, IL-10, IL-12p40, IL-12p70, IL-15, IL-18, IL-2, IL-6, TNFα, GM-CSF, CCL3 (MIP-1α), and IL-27 expression of vaginal lavages from HSV-1-infected mice untreated or IFNβ-treated relative to naive mice. Mean values normalized to naive mice from 10 (HSV-1), 13 (HSV-1 + IFNβ), and 5 (naive) mice are shown.

Initial evaluation of different murine type I IFNs as potential therapeutics for genital HSV-1 infections revealed a faster reduction of local viral loads following IFNβ treatment (**Figure 1B**). On day 8 *p.i*., only minor immunomodulatory effects of IFNβ were detectable in the spleen, but not within the vaginal tract, the local site of infection. Thus, we further assessed the respective modulations of local and systemic immune responses after IFNβ treatment at an earlier timepoint during HSV-1 infection, when the virus is still replicating within the vaginal tract. We therefore infected female mice with HSV-1 and treated these mice on days 2 and 3 *p.i.* with IFNβ according to the previous experiments (**Figure 1A**). On day 4 *p.i*., when IFNβ treatment induced a significant reduction in viral loads (**Figure 1B**), mice were sacrificed and viral loads as well as splenic and local immune responses in the vaginal tract were determined. Again, we observed a strong reduction in viral loads on day 4 *p.i.* (**Figure 4A**). Furthermore, we also analyzed 14 different cytokines and chemokines in the vaginal lavages at 4 dpi. As depicted in **Figure 4B**, IL-27 was not induced during HSV-1 infection, whereas all other tested cytokines and chemokines were induced during HSV-1 infection (mean fold change (FC) between 1.7 for IL-15 and 420.8 for CCL5 relative to naive mice). Mice treated with IFNβ showed reduced levels of CCL5, CCL3, IL12p40, IFNγ, and IL-6 compared to untreated, HSV-1-infected mice, suggesting that either the reduced viral titers in IFNβ-treated mice or IFNβ itself inhibited inflammation within the vaginal tract.

**Figure 5:**
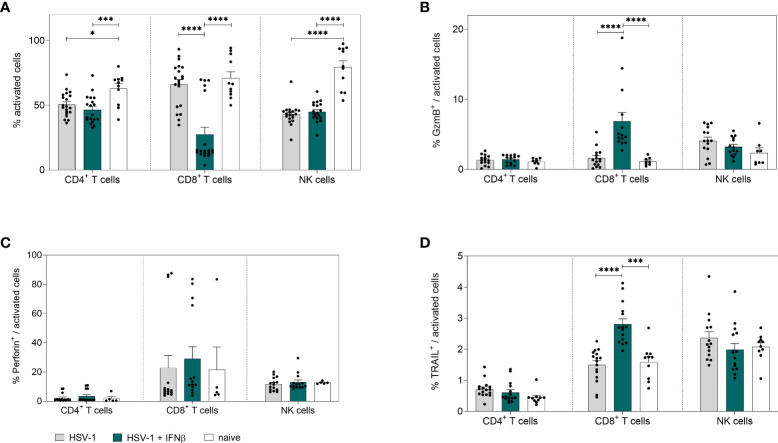
Peripheral immune responses in the spleen on day 4 post HSV-1 infection. Female C57BL/6 mice were infected with 1×10^7^ TCID_50_ of HSV-1 F for four days or left uninfected (naive control). Mice were treated with 8000 U of murine IFNβ for two consecutive days starting on day 2 *p.i.* or left untreated (HSV-1). On day 4 *p.i*., splenic lymphocytes were isolated from dissected spleens. For flow cytometric analysis, isolated cells were stained for respective cell surface and intracellular markers. **(A)** Frequencies of activated CD4^+^ T cells, CD8^+^ T cells and NK cells as well as frequencies of **(B)** GzmB^+^, **(C)** Perforin^+^ and **(D)** TRAIL^+^ activated cell subsets are shown. Data points represent individual mice. Bars indicate the mean values ± SEM for each group (HSV-1 *n* = 14-20 mice, HSV-1 + IFNβ *n* = 14-19 mice, naive *n* = 5-11 mice). Statistically significant differences among all study groups were analyzed with the 2way ANOVA and Tukey’s multiple comparison test and are depicted by *P < 0.05, ***P < 0.001, ****P < 0.0001.

To further scrutinize whether the treatment with IFNβ also modulates immune cell functions apart from its direct antiviral activity, we analyzed innate and adaptive immune cell responses in the spleen. As depicted in **Figure 5A**, treatment with IFNβ significantly reduced the frequencies of activated CD8^+^ T cells, which is comparable to the previous results on day 8 *p.i*. (**Figure 2A**). Interestingly, already early during HSV-1 infection, percentages of cytotoxic CD8^+^ T cells expressing GzmB or TRAIL were significantly elevated after IFNβ treatment compared to untreated controls (**Figures 5B, D**), whereas no effects on splenic cytotoxic and cytokine-producing CD4^+^ T cells or NK cells were found at that time point (**Figures 5B-D**; **Supplementary Figure 4**).

**Figure 6:**
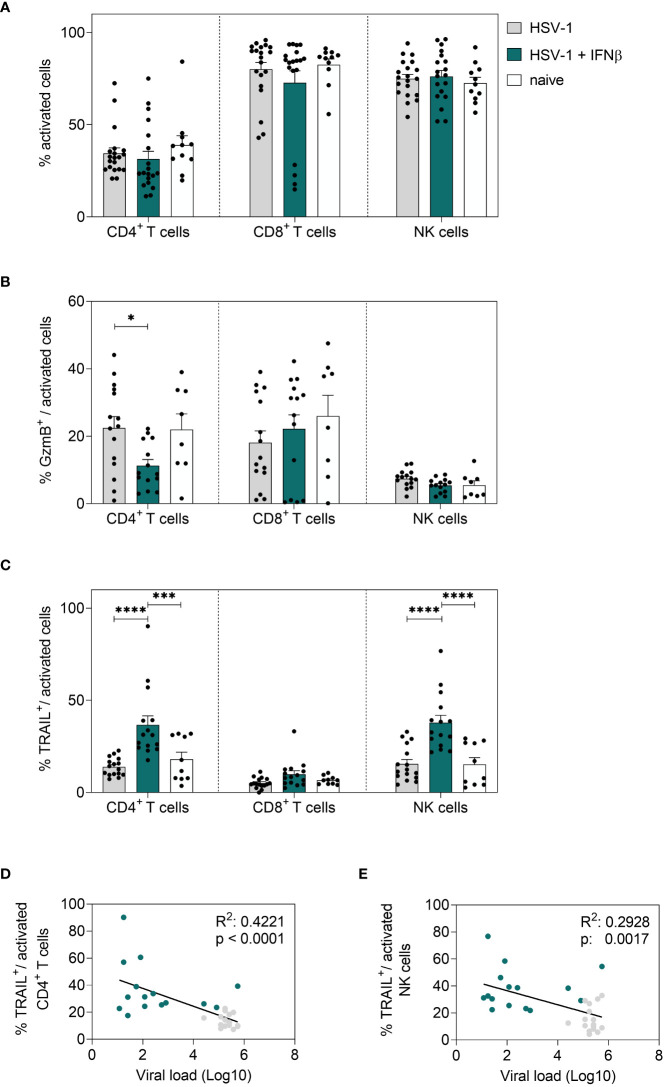
Local immune responses in the vaginal tract on day 4 post HSV-1 infection. Female C57BL/6 mice were infected with 1×10^7^ TCID_50_ of HSV-1 F for four days or left uninfected (naive control). Mice were treated with 8000 U of murine IFNβ for two consecutive days starting on day 2 *p.i.* or left untreated (HSV-1). On day 4 *p.i*., vaginal leukocytes were isolated from dissected vaginal tracts. For flow cytometric analysis, isolated cells were subsequently stained for respective cell surface and intracellular markers. **(A)** Frequencies of activated CD4^+^ T cells, CD8^+^ T cells and NK cells, frequencies of **(B)** GzmB^+^ and **(C)** TRAIL^+^ activated cell subsets are shown. Data points represent individual mice. Bars indicate the mean values ± SEM for each group (HSV-1 *n* = 15-20 mice, HSV-1 + IFNβ *n* = 14-19 mice, naive *n* = 8-11 mice). Statistically significant differences among all study groups were analyzed with the 2way ANOVA and Tukey’s multiple comparison test and are depicted by *P < 0.05, ***P < 0.001, ****P < 0.0001. Correlation of frequencies of TRAIL^+^ activated CD4^+^ T cells **(D)** or frequencies of TRAIL^+^ activated NK cells **(E)** and the viral loads (Log10) at 4 dpi of IFNβ-treated, HSV-1-infected (teal dots) and untreated, HSV-1-infected (grey dots) mice. Data points represent individual mice. Statistically significant differences among all study groups were analyzed with simple linear regression.

Next, we also determined IFNβ-mediated effects on local immune responses in the vaginal tract. Corresponding to 8 dpi, no changes in the percentages of activated T cells or NK cells in the vaginal tract were observed earlier (**Figure 6A**); however, a strong increase in frequencies of cytotoxic CD4^+^ T cells and NK cells (TRAIL^+^) was detected (**Figure 6C**). No changes in cytotoxic CD8^+^ T cells could be observed upon IFNβ stimulation. Moreover, the overall frequencies of CD8^+^ T cells at that early time point were rather low (**Figure 6B, C** and data not shown). Also, B cell and DC responses in the vaginal tract were not affected by IFNβ treatment in comparison to untreated HSV-1-infected mice (data not shown). In **Figure 4** we recognized separate patterns in the viral loads with 7 out of 28 mice in the IFNβ-treated group still showing high viral loads, whereas 6 out of 35 mice in the untreated group had low viral loads. Thus, we determined if those mice with high viral loads after IFNβ treatment failed to induce an efficient immune response at 4 dpi in the vaginal tract. We observed a negative correlation of the percentage of TRAIL-expressing CD4^+^ T cells and NK cells with viral loads, indicating that lower frequencies of TRAIL-expressing cells might drive higher viral loads (**Figures 6D, E**).

In conclusion, we demonstrated that IFNβ strongly reduced HSV-1 viral titers in the vaginal tract, which was not observed after treatment with IFNα1, IFNα4, or IFNα11. In addition to its direct antiviral activity, IFNβ strongly increased cytotoxic T and NK cell responses in the spleen and in the vaginal tract early during HSV-1 infection. Importantly, elevated frequencies of TRAIL-expressing NK and CD4^+^ T cells are required for early viral control.

## Discussion

Herpes simplex virus infections are usually associated with self-limiting, orofacial or genital lesions. However, they present an increasing incidence of resistance to conventional treatment agents, thus constituting a major health problem especially for immunocompromised hosts, who easily develop life-threatening sequelae due to resistant virus strains (Stránská et al., 2005; Fatahzadeh and Schwartz, 2007; Sauerbrei et al., 2011). Additionally, neither a prophylactic vaccine nor a curing therapeutic is available so far. Thereby, previous attempts for the development of new antiviral agents have been mainly hampered by the complexity of HSV-specific immune responses, assuming the induction of both innate as well as adaptive immunity to be required for sufficient long-term protection (Koelle and Corey, 2008; Belshe et al., 2012).

In this regard, type I IFNs are known to induce a broad spectrum of cellular responses, including antiviral, immunomodulatory and antiproliferative activities to control viral infections. As part of the innate immunity, they activate early immune responses, but also modulate and regulate adaptive immune cell functions (Hervas-Stubbs et al., 2011; Teijaro, 2016). During HSV-1 and HSV-2 infections several type I IFN-related activities have been identified, which promote the impairment of viral replication and the establishment of an antiherpetic environment (Danastas et al., 2020; Feng et al., 2021). Conversely, HSV has been verified to specifically circumvent the type I IFN system by interfering with respective signaling events, thus assuring the development of latency and further reactivation (Koelle and Corey, 2008; Xu et al., 2019; Danastas et al., 2020).

Concerning this close interplay of immunity and viral evasion strategies, the question arises whether additional, exogenous type I IFNs can counteract the HSV-specific evasion and further enhance the properties of endogenously produced type I IFNs. In our study, we selected 4 different type I IFN subtypes (IFNα1, IFNα4, IFNα11, and IFNβ) and applied them therapeutically to HSV-1-infected mice. The treatment started at 2 dpi, when the vaginal infection was already established and viral loads peaked. Only exogenous application of IFNβ resulted in significantly reduced viral titers, whereas treatment with IFNα1, IFNα4, or IFNα11 had no effect on viral replication (**Figure 1B**). So far, clinical trials evaluating the treatment of oral and genital HSV infections with the clinically approved human IFNα2 could already reveal an antiviral efficacy of exogenous type I IFN leading to decreased symptom duration and severity as well as a reduction of recurrence incidence, but without reaching complete prevention of infection and latency (Jones et al., 1976; Kuhls et al., 1986; Ho, 1990; Cardamakis et al., 1998). In this regard, previous *in vivo* studies with recombinant type I IFN subtypes as well as plasmids expressing different type I IFN genes examined a subtype-specific antiherpetic potential, thus assuming other type I IFN subtypes than human IFNα2 to be more efficient for HSV-1 and HSV-2 treatment (Härle et al., 2001; Austin et al., 2006; Giraldo et al., 2020). Hence, within the present study different type I IFN subtypes were assessed for their therapeutic potential in genital HSV-1 infections *via* a murine *in vivo* model, thereby focusing not only on the overall antiviral efficacy, but also on potential qualitative subtype-specific differences in underlying immune responses. In general, we observed an antiherpetic activity of IFNα1, IFNα2, IFNα4, IFNα5, IFNα6, IFNα9, IFNα11 and IFNβ *in vitro* (**Supplementary Figure 1**). Interestingly, those IFNα subtypes, which significantly reduced viral replication around 10-fold *in vitro* (IFNα1, IFNα4, and IFNα11), did not inhibit viral replication *in vivo* (**Figure 1B**) and their immunomodulatory potency during HSV-1 infection was rather low (**Figures 2A**, **3A** and **Supplementary Figure 3**). In contrast, IFNβ with the lowest antiviral activity against HSV-1 *in vitro* strongly reduced viral loads already after two IFN doses *in vivo*, suggesting that apart from its direct antiviral effect, IFN-regulated immune responses are required for efficient HSV-1 control. Additionally, in the *in vitro* experiments type I IFNs were added before virus infection, that cells become alerted towards an antiviral state and antiviral effectors can be transcribed or even translated prior to viral infection. In the *in vivo* experiments, we added the IFNs when HSV-1 infection was already established suggesting the timing of the IFN treatment might also influence the antiviral effect of IFNs. Others reported similar direct antiviral activities in L929 cells transfected with different murine type I IFN transgenes, but they observed the strongest reduction *in vitro* with IFNβ and IFNα4 as most effective IFNs against HSV-1 (Härle et al., 2002). In addition, *in vivo* evaluation of these transgenes verified an increased reduction in the HSV-2 dependent mortality for IFNα1 and IFNβ (Härle et al., 2002). Interestingly, it was also reported that IFNγ, which has a very low antiviral activity against HSV-1 (Harle et al., 2002), synergizes with IFNα and IFNβ to control HSV-1 infection *in vitro* and pre-treatment with IFNβ and IFNγ reduced the numbers of latent HSV-1 genomes in ocular HSV-1 infection *in vivo* (Sainz and Halford, 2002). These data suggest, that IFNγ, which is mainly produced by T cells markedly improves type I IFN-mediated control of HSV-1. In our study we did not detect a significant increase in IFNγ expressing T or NK cells (**Figure 2C**; **Supplementary Figures 3A, C, E**; **Supplementary Figure 4A**), although we observed an induction of IFNγ in the vaginal lavages at 4 dpi which might be produced by other important immune cell subsets within vaginal tract like granulocytes, monocytes, or macrophages. Other important members of the type I IFN family (IFNϵ, IFNκ) were already characterized during viral infections like HSV-1/2, Zika virus or human papillomavirus (HPV) in the vaginal tract (Reiser et al., 2011; Fung et al., 2013; Habiger et al., 2016; Woodby et al., 2018; Mungin et al., 2022) or in HSV-1-infected keratinocytes (Li et al., 2020; Kalke et al., 2022). Mice deficient in IFNϵ, which is constitutively expressed by epithelial cells in the female reproductive tract, were highly susceptible to HSV-2 (Fung et al., 2013).

Although we saw a direct antiviral effect of IFNα1, IFNα4, and IFNα11 *in vitro*, we did not observe any antiviral effect of these IFNα subtypes *in vivo*. In this concern, type I IFNs are also known to hold additional proviral functions either by downregulating protective immune responses or by excessive stimulation of inflammatory signals including pro-inflammatory cytokines like IFNγ, IL-6, IL-9, and granulocyte-colony stimulating factor (G-CSF) as well as chemoattractants like Eotaxin, IFNγ-induced protein 10 (IP-10), monocyte chemoattractant protein 1 (MCP-1) and macrophage inflammatory protein 1β (MIP-1β) (Davidson et al., 2014; Cheng et al., 2017). Here, we observed reduced levels of pro-inflammatory cytokines upon IFNβ-stimulation (**Figure 4B**), which might be either directly downregulated by IFNβ or the reduced viral loads have not triggered an inflammatory state in the female reproductive tract.

Nevertheless, analysis of viral loads alone does not confer an adequate comprehension of the antiherpetic potency of type I IFN subtypes and the here observed differences between subtypes. Therefore, the corresponding local immunity of vaginal tracts as well as systemic responses in spleens were further analyzed on days 4 and 8 *p.i*., aiming to decipher the activation or effector functions of different cell types. From previous studies of the HSV-induced immunity it is known that a variety of cell types like NK or T cells are involved, relying on fine-tuned interactions *via* cytokines, chemokines, or co-stimulatory molecules (Chew et al., 2009; Truong et al., 2019). NK cells represent the predominant cell population of the innate immunity, that mediates cytotoxic and cytolytic attacks without prior antigen exposure, hence playing an important role for numerous viral infections. In this concern, NK cell-depletion-studies in HSV-1-infected mice could further reinforce the indispensable role of NK cells for the HSV-specific immunity resulting in increased viral loads and mortality of depleted mice (Habu et al., 1984; Nandakumar et al., 2008). Furthermore, they also reported that IFN treatment of HSV-1-infected mice increased the survival, which was not the case in NK cell-depleted mice (Habu et al., 1984), and the treatment with a mixture of type I IFNs strongly improved NK cell cytotoxicity. As typical effector cells of the adaptive immunity, CD8^+^ T cells are characterized by an extensive expansion as well as differentiation into either cytotoxic or suppressive cell subsets. Following cross-priming with DCs, CD8^+^ T cells were reported to interfere with the replication of HSV-1 and HSV-2 by releasing different cytokines or cytotoxic molecules and further evolve special memory functions which are assumed to contribute to a protective immunity (Shin et al., 2016; Vahed et al., 2019). Besides CD8^+^ T cells, also CD4^+^ T cells are detected during HSV infection. CD4^+^ T cells are predominantly known for their regulatory or helper functions, thereby indirectly influencing the host defense. CD4^+^ T cells were observed to hold cytotoxic activities and mediate the release of certain cytokines during HSV infections (Yasukawa et al., 1991). It was previously reported that *in vivo* treatment with plasmids encoding *IFNA1*, local and distal CD4^+^ and CD8^+^ T cell effector functions are required to antagonize ocular HSV-1 infections (Noisakran and Carr, 2000) and CD8^+^ T cells are important for HSV-2 control (Austin et al., 2006), whereas IFNα1 treatment had no effect on NK cell cytotoxicity (Härle et al., 2001). Comprehensive and detailed investigations on IFN-mediated immune responses during vaginal HSV-1 infection are still lacking. The present study of genital HSV-1 infections could verify the induction of NK cell as well as CD4^+^ and CD8^+^ T cell immune responses following IFNβ treatment at both local and peripheral sites (**Figures 3**, **5**, **6**; **Supplementary Figures 2**–**4**). Here late infection time points, where local virus replication was already absent, were characterized by a significant increase of activated NK cells, but in turn also by suppression of activated CD8^+^ T cells as well as the expression of cytokines and cytotoxic molecules. In contrast at very early time points during infection, treatment with IFNβ could elevate those cytotoxic effector functions for CD4^+^ T cells and NK cells especially at the vaginal tract, thereby negatively correlating with the superior reduction of local viral loads in IFNβ-treated mice. Hence, these findings implicate a pivotal role of the early, local cytotoxicity mediated by IFNβ-stimulated NK and T cell populations for sufficient HSV-1 control. Correspondingly, previous studies on genital HSV-2 infections have also reported a particular upregulation of IL-15 within the genital mucosa (**Figure 4B**), that was closely associated with an enhanced activation of cytotoxic NK cells, thus supporting the indispensable role of NK cell responses for HSV immunity (Gill et al., 2011).

The variance among the different type I IFN subtypes tested in this study is in accordance with the concept of qualitative differences among type I IFNs (Lavender et al., 2016; Dickow et al., 2019; Guo et al., 2020; Chen et al., 2021; Schuhenn et al., 2022). Each subtype was assessed for its antiviral efficacies and these differences were due to induction of specific local and systemic immune responses, that altered HSV-1-induced immunity. In this concern, it would be revelatory to decipher specific properties of the remaining type I IFN subtypes as well as potential synergistic effects of certain subtypes which possibly contribute to a protective HSV-1 immunity. Furthermore, longitudinal studies to evaluate their long-term efficacy for recurrent infections as well as their impact on latency establishment have to be further validated.

## Data availability statement

The original contributions presented in the study are included in the article/**Supplementary Material**. Further inquiries can be directed to the corresponding author.

## Ethics statement

The animal study was reviewed and approved by North Rhine-Westphalia State Agency for Nature, Environment and Consumer Protection (LANUV).

## Author contributions

KS and AK conceived the study. YS and MS substantially contributed to the acquisition and analysis of the data. JW, ELS, MA and LB provided reagents and performed experiments. KS wrote the original manuscript and all authors edited and approved the final manuscript.

## Funding

This work was supported by the DFG RTG 1949 to KS. We acknowledge support by the Open Access Publication Fund of the University of Duisburg-Essen.

## Conflict of interest

The authors declare that the research was conducted in the absence of any commercial or financial relationships that could be construed as a potential conflict of interest.

## Publisher’s note

All claims expressed in this article are solely those of the authors and do not necessarily represent those of their affiliated organizations, or those of the publisher, the editors and the reviewers. Any product that may be evaluated in this article, or claim that may be made by its manufacturer, is not guaranteed or endorsed by the publisher.
