# Supplementary figures and images for: Superior antiviral activity of IFNβ in genital HSV-1 infection
Source: Front Cell Infect Microbiol. 2022 Oct 17;12:949036. doi: 10.3389/fcimb.2022.949036 (PMC9618724; doi:10.3389/fcimb.2022.949036)

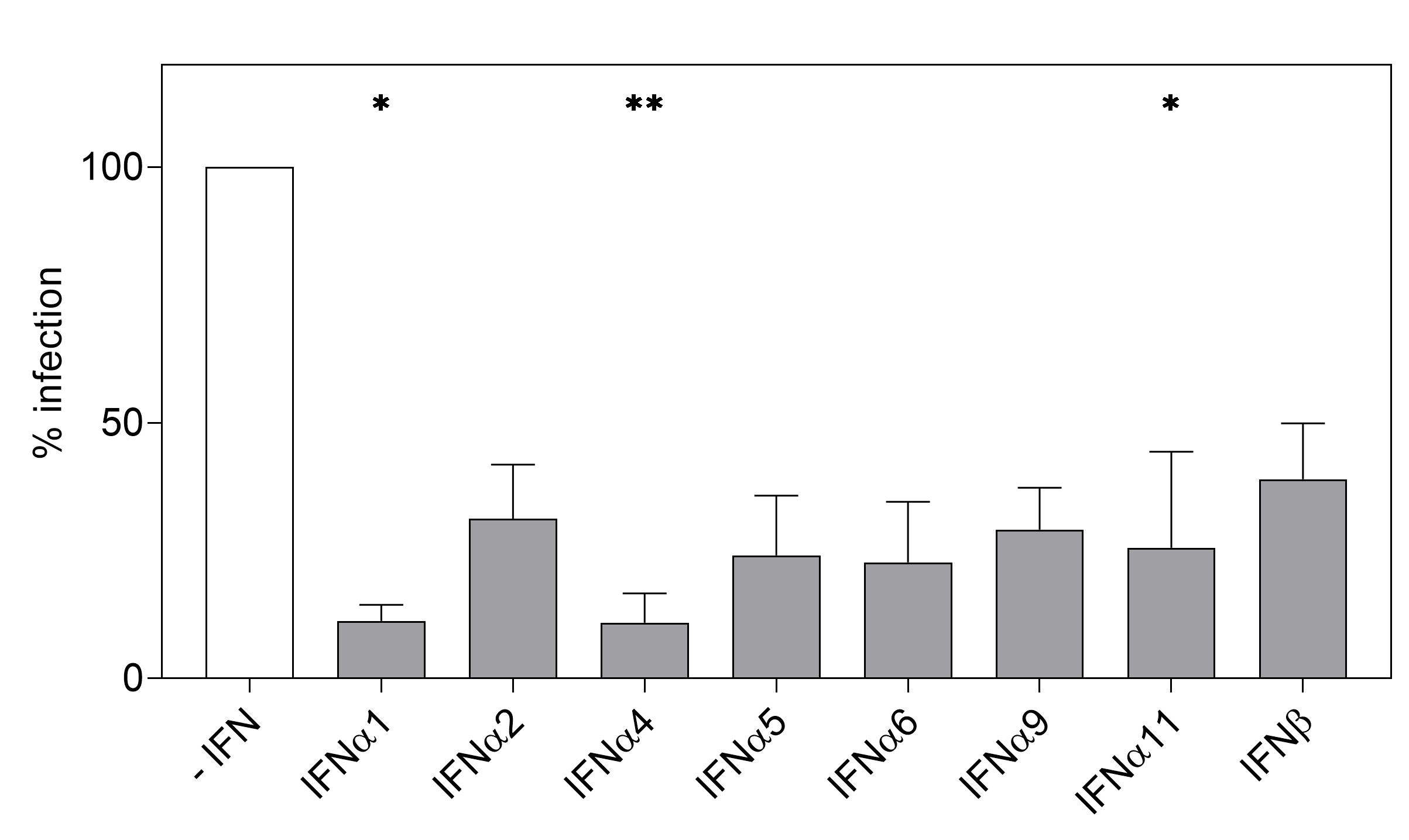

Supplement: Supplementary file 1 [file Image_1.tif]

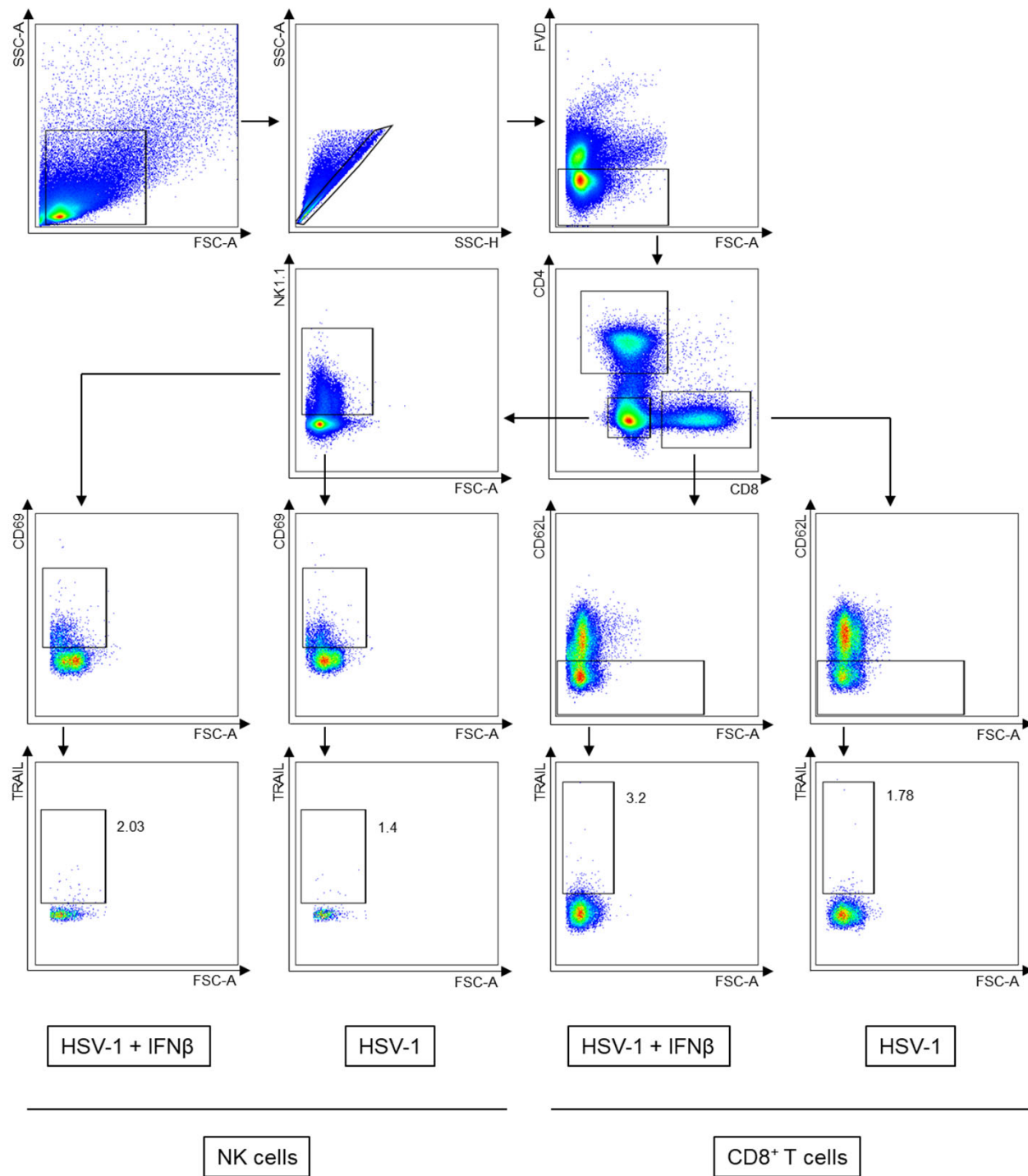

Supplement: Supplementary file 2 [file Image_2.pdf]

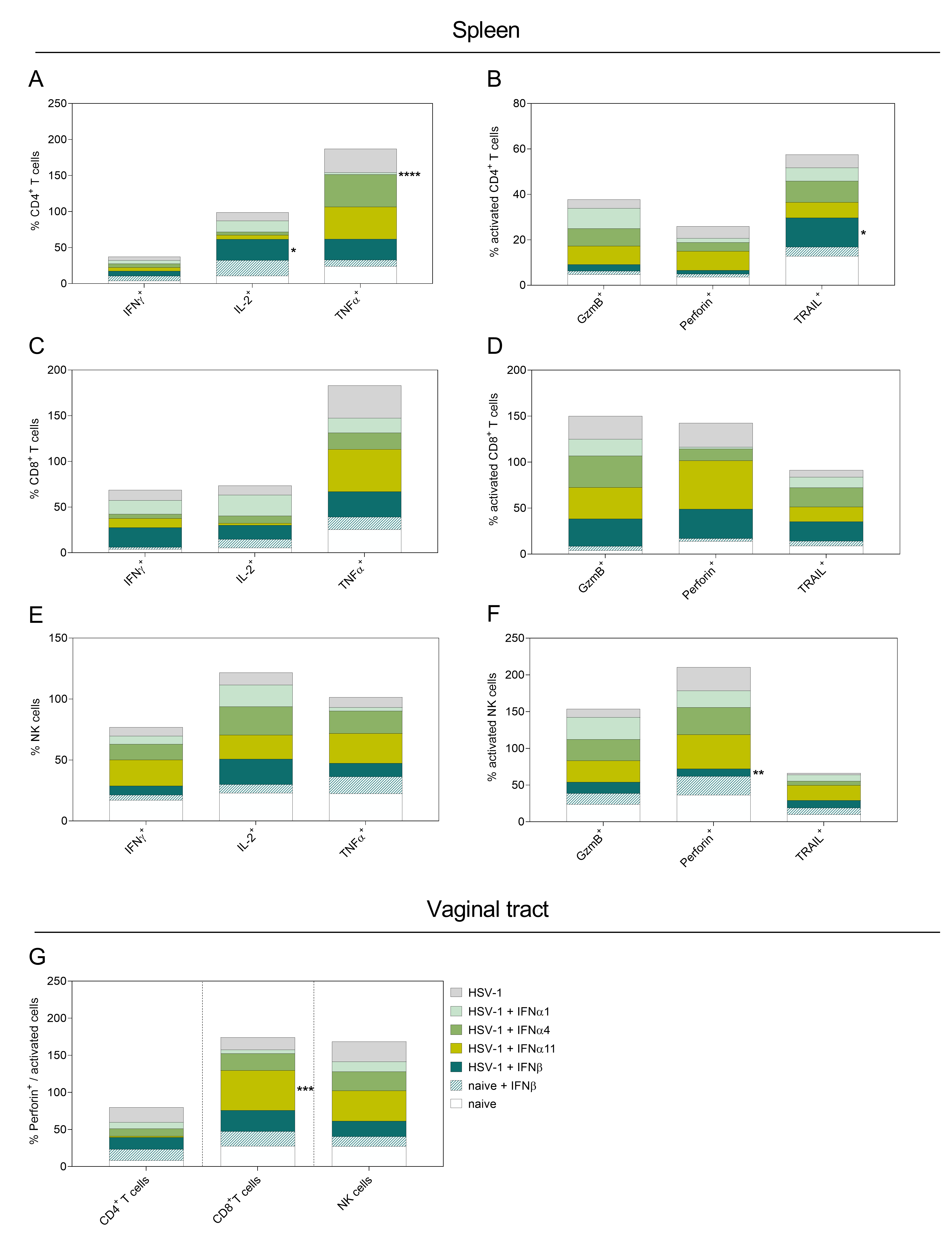

Supplement: Supplementary file 3 [file Image_3.tif]

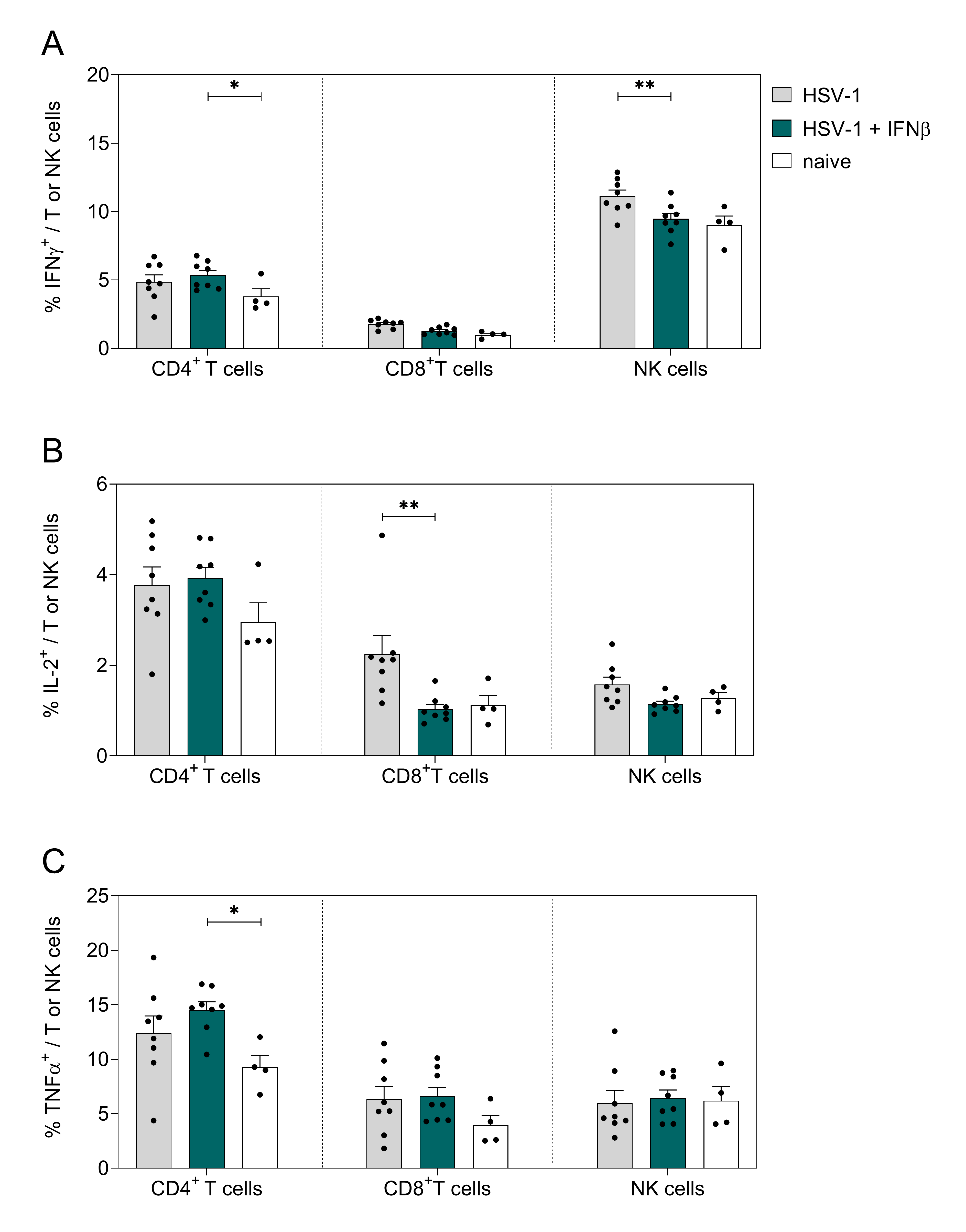

Supplement: Supplementary file 4 [file Image_4.tif]
